# Risk Factors of Febrile Status Epilepticus

**Published:** 2019

**Authors:** Reza SHARAFI, Afagh HASSANZADEH RAD, Vahid AMINZADEH

**Affiliations:** 1 Pediatric Growth Disorder Research Center, 17 Shahrivar HospitalSchool, of Medicine , Rasht, Iran

**Keywords:** Febrile status epilepticus, Children, Risk factor

## Abstract

**Objective:**

We aimed to investigate the risk factors of febrile status epilepticus (FSE) in children.

**Materials & Methods:**

This analytic case-control study was conducted on all patients’ records with first febrile seizure (FS) admitted to 17 Shahrivar Hospital, Rasht, Iran during 2007-2014. Cases were children aged 6 to 60 months with FSE and controls were children with complex and simple FS. Data were gathered using a checklist including age, sex, type of milk consuming during first year, temperature, the interval between fever and seizure, family history of epilepsy and febrile seizure, and prematurity. Data were analyzed using Chi-square in SPSS 19.

**Results:**

Overall, 756 patients with FS participated including 39 patients with FSE, 194 complex febrile seizure (CFC) and 523 simple febrile seizure (SFC). Most of the patients (57.8%) experienced seizure with low-grade fever (<39 °C). The mean age in SFC group was significantly higher than FSE patients (*P*<0.05). A significant relation was noted between groups regarding body temperature during seizure (*P*=0.006), family history of FS (0.029), family history of epilepsy (*P*=0.042) and the premature birth (*P*=0.023). Significant relation was noted between FSE and CFC groups regarding body temperature during seizure (*P*=0.004), family history of FS (0.011), family history of epilepsy (*P*=0.037), and the premature birth (*P*=0.025) between FSE and CFC groups.

**Conclusion:**

Considering risk factors of FSE including low body temperature, lower age, family history of FS and epilepsy, and premature birth is mandatory.

## Introduction

Febrile seizure (FS) is the most common seizure disorder during childhood which occurs in 2%-5% of children. It is defined as the occurrence of seizure with at least 38 °C rectal fever in children aged 6-60 months with the absence of central nervous system infection, electrolyte imbalance, and previous afebrile seizure (1). It is classified into complex and simple types. Complex type is determined by focal seizures or occurred at least 2 times during febrile illness or seizure with more than 15 min length. It happens in approximately 30% of FS (1).

Clinicians indicate FSE if one seizure lasts for more than 30 min or successive seizures without consciousness. It is noted as an important issue in pediatric neurology (2). It occurs with relatively high prevalence and has high likelihood for epilepsy and death. It causes in 5% of FS and involves in 25% of all types of childhood status epilepticus (3). On the other hand, it is the most common type of childhood status epilepticus especially in children aged less than 3 years (4).

Although developmental delay, presence of child in day care centers, family history of FS and epilepsy, prematurity, and low birth weight has been determined as risk factors of first FS, there is no definite knowledge regarding risk factors of FSE yet (5). Some risk factors of first FS may be indicated as risk factors of FSE. Moreover, FSE can be related to future epilepsy. Therefore, possible risk factors following increased rate of epilepsy after FS such as developmental disability, focal febrile seizure, family history of epilepsy and low interval between fever and seizure (less than 1 h) may increase risk of FSE (6).

As the FSE is an important, neurological emergency and may cause considerable increase in epilepsy, mesial temporal sclerosis and refractory temporal lobe epilepsy (7), and also prolonged FSE can increase the risk of renal damage (8) and meningitis (9), therefore, we aimed to investigate the risk factors of FSE in children. 

## Materials & Methods

This analytic case-control study was conducted on all patients’ records with first FS admitted to 17 Shahrivar Hospital, Rasht, north part of Iran during 2007-2014,. Cases were children aged 6 to 60 months with FSE and controls were children with complex and simple febrile seizures (SFS). Children with developmental disorders were excluded. 

Ethical approval was obtained from Guilan University of Medical Sciences and informed consent was taken from the parents. 

Data were gathered using a checklist including age, sex, type of milk consuming during first year, temperature, the interval between fever and seizure, family history of epilepsy and febrile seizure, and prematurity. Data were reported using descriptive statistics (number, percent, mean, standard deviation) and analyzed by Chi-square in SPSS 19 (Chicago, IL, USA). 

## Results

Overall, 756 patients with FS participated including 39 patients with FSE, 194 complex febrile seizure (CFC) and 523 SFC. The mean age was 21.2±10.9 months. Just 29.8% of patients exclusively breastfed. Most of the patients (57.8%) experienced seizure with low-grade fever (<39 °C). The majority of seizures (60%) occurred during 1-24 h between fever onset and the occurrence of seizure. 116 (15.3%) patients prematurely born (Table 1).

Results showed no significant relationship between sex and type of feeding in patients with FSE and SFC (*P*>0.05). There was significant difference between these groups regarding age. The mean age in SFC group was significantly higher than FSE patients (*P*<0.05). A significant relation was noted between groups regarding body temperature during seizure (*P*=0.006), family history of FS (0.029), family history of epilepsy (*P*=0.042) and the premature birth (*P*=0.023) (Table 2).

In addition, results mentioned no significant relationship between sex and type of feeding in patients with FSE and CFC. 

In addition, significant difference between FSE and CFC groups was mentioned regarding age. The mean age in FSE group was significantly higher than CFC patients (*P*<0.05). Significant relation was noted between groups regarding body temperature during seizure (*P*=0.004), family history of FS (0.011), family history of epilepsy (*P*=0.037), and the premature birth (*P*=0.025) (Table 3). 

**Table 1:** Demographic and baseline characteristics of participants

Variable	Number	Percent
Sex		
Boy Girl	381 375	50.3 49.7
Age groups		
1-18 months >18 months	346 410	45.7 54.3
Type of feeding		
Exclusive breastfeeding Formula feeding	226 530	29.8 71.2
Body temperature during FS		
<39 °C ≥39 °C	437 319	57.8 42.2
The interval between fever onset and the occurrence of seizure		
<1 h 1-24 h >24 h	157 454 145	20.7 60 19.3
Family history of febrile seizure		
Yes No	201 555	26.5 73.5
Family history of epilepsy		
Yes No	27 729	3.5 96.5
Premature birth		
Yes No	116 640	15.3 84.7

**Table 2:** Comparing characteristics of FSE and SFC groups

Variable	FSE group N (%)	SFC group N (%)	*P*-value
Sex			
Boy Girl	20(51.3) 19(48.7)	262(51) 261(49)	0.843
Age groups			
1-18 months >18 months	26(66.7) 13(33.3)	203(38.8) 320(61.8)	0.013
Type of feeding			
Exclusive breastfeeding Formula feeding	8(20) 31(80)	163(31.6) 360(68.4)	0.316
Body temperature during FS			
<39 °C ≥39 °C	34(87.1) 281(53.7)	55(12.9) 242(46.3)	0.006
The interval between fever onset and the occurrence of seizure			
<1 hour 1-24 hour >24 hour	7(18) 101(19.3) 29(74.3)	315(60.2) 3(7.7) 107(20.5)	0.741
Family history of febrile seizure			
Yes No	10(25.6) 148(28.3)	29(74.4) 375(71.7)	0.029
Family history of epilepsy			
Yes No	2(3.7) 21(2.4)	37(96.3) 721(97.6)	0.042
Premature birth			
Yes No	7(17.8) 60(11.4)	32(82.2) 463(88.6)	0.023

**Table 3:** Comparing characteristics of FSE and CFC groups

Variable	FSE group N (%)	CFC group N (%)	*P*-value
Sex			
Boy Girl	20(51.3%) 19(48.7%)	99(50.1%) 95(49.9%)	0.734
Age groups			
1-18 months >18 months	26(66.7) 13(33.3)	117(60.3) 77(39.7)	0.023
Type of feeding			
Exclusive breastfeeding Formula feeding	8(20) 31(80)	55(28) 139(72)	0.324
Body temperature during FS			
<39 °C ≥39 °C	34(87.1) 281(53.7)	122(62.8) 72(37.3)	0.004
Family history of febrile seizure			
Yes No	10(25.6) 148(28.3)	43(22.1) 151(77.9)	0.011
Family history of epilepsy			
Yes No	2(3.7) 21(2.4)	4(2.7) 112(97.3)	0.037
Premature birth			
Yes No	7(17.8) 60(11.4)	49(25.2) 145(74.8)	0.025

## Discussion

FSE is the most common cause of SE especially in children aged less than 3 yr, therefore assessing the risk factors of FSE in patients with FS is necessary for on-time prevention and treatment. Age, degree of temperature, formula feeding, and history of prematurity can be noted as risk factors of FSE. 

The frequency of FSE, SFC, and CFC was slightly higher in boys, but no statistically significant relation was noted regarding sex. This result was consistent with previous study where 52% of the participants with FSE were boys (10). This was consistent with the idea in which male sex could be a minor risk factor for recurrence of FC.

The mean age in patients was reported as 21.2±10.9 months which was similar. Most of the patients in FSE and CFC groups aged 1-18 months and 61.8% of patients with SFC aged >18 months. There was a significant difference between groups regarding age which was similar to previous study where FSE occurred in lower aged patients and age less than 16 months was a considerable risk factor for CFC (6).

Most of the patients in FSE group (87.1%) had low-grade fever (<39 °C) during the seizure and significant relation was noted between the degree of temperature and the occurrence of FSE which was similar another study reported low-grade fever during FSE in the majority of patients (6). 

Regarding the probable higher risk of epilepsy in children with premature birth, investigators assessed premature birth as a risk factor. In FSE, SFC and CFC groups, 17.8%, 11.4%, and 25.2% of patients mentioned the history of premature birth, respectively and significant difference was noted between groups. Premature birth was mentioned as a risk factor for the occurrence of FSE (11). 

The interval between fever and the occurrence of seizure in most of the patients in all 3 groups was 1-24 h (74.3% FSE, 60.2% SFC, 56.7% CFC), but no significant difference was noted between groups. Hestroffer et al mentioned consistent results (6). As febrile status increased the chance of epilepsy and instead the lower duration between fever and seizure has been mentioned as a known risk factor for epilepsy, our finding was inconsistent with the literature and further investigations may be recommended.

**In conclusion, **the occurrence rate of FSE was higher in lower body temperature (≤39 °C), lower age group (≤18 months), positive history of FS and epilepsy, and history of premature birth. Although there was no significant relation between formula feeding, the interval between fever and seizure, and sex in groups, the occurrence of FSE was higher in boys, fed by formula at 1-24 h after fever. Further investigation which assesses these factors can be recommended. 
